# p73 to the rescue: Role of RPL26

**DOI:** 10.18632/oncotarget.14383

**Published:** 2016-12-30

**Authors:** Xiao-Xin Sun, Mu-Shui Dai

**Affiliations:** Departments of Molecular and Medical Genetics, School of Medicine, Portland, OR, USA; The OHSU Knight Cancer Institute, Oregon Health and Science University, Portland, OR, USA

**Keywords:** RPL26, p73, p53, ribosome biogenesis, ribosomal stress

For the past decade, a group of ribosomal proteins (RPs) have emerged as key mediators of the p53 signaling in response to ribosomal stress, which is induced by perturbation of ribosome biogenesis [1, 2]. These RPs include large subunit proteins RPL5, RPL11, RPL23, RPL26, RPL4, RPL6 and small subunit proteins RPS7, RPS27, RPS27a, RPS14, RPS25, etc [1-3]. When overexpressed or upon ribosomal stress, these RPs bind to MDM2 and repress MDM2-mediated p53 ubiquitination and proteasomal degradation, thereby stabilizing p53 and inducing cell cycle arrest. p53 also inhibits ribosome biogenesis by repressing the transcription of rRNA, RPs and tRNAs catalyzed by RNA Pol I, II and III, respectively. Thus, the RP-MDM2-p53 pathway functions to tightly coordinate cell cycle progress (cell proliferation) with ribosome biogenesis (cell growth) and plays an important role in maintaining normal cell homeostasis and preventing cell transformation. Mice with a knock-in of RPL5 and RPL11 binding defective MDM2 mutant (C305F) displayed a specific defect in p53 signaling in response to ribosomal stress, but not DNA damage [4], validating the critical role for the RP-MDM2-p53 pathway *in vivo*. In addition, RPs also regulate cell growth and proliferation via p53-independent mechanisms, including their direct inhibition of c-Myc, a master regulator of gene transcription and ribosome biogenesis, as well as promoting E2F1 degradation by releasing E2F1 from MDM2 binding [1, 2].

**Figure 1:**
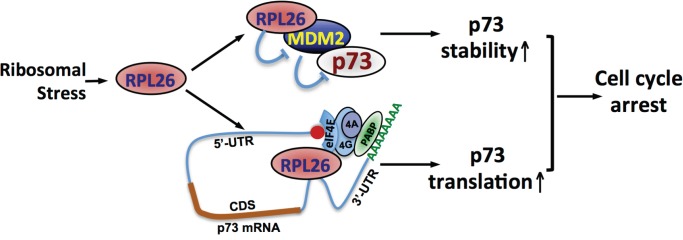
RPL26 induces p73 expression via both stabilizing p73 and promoting p73 translation

Interestingly, the RP-MDM2 regulation does not just activate p53. In this issue, Zhang et al [5] identified RPL26 as a novel positive regulator of p73, a p53 family tumor suppressor protein. Knockdown of RPL26 reduced the levels of p73 whereas overexpression of RPL26 increases the levels of p73 in various cancer cell lines regardless of the status of p53. RPL26 has been previously shown to induce the levels of p53 by two mechanisms. On one hand, RPL26, like other RPs, binds to MDM2 and inhibits MDM2-mediated p53 ubiquitination and degradation, thereby stabilizing p53 [6]. On the other hand, RPL26 binds to a specific dsRNA structure formed by base pairing and looping between the 5’-UTR and 3’-UTR of p53 mRNA and promotes p53 translation [7]. Similarly, RPL26 also regulates p73 protein stability via inhibiting MDM2. Overexpression of RPL26 significantly prolonged the half-life of p73. Knocking out MDM2 by CRISP-cas9 technology increased p73 stability in multiple cell lines, but partially attenuated the reduction of p73 by RPL26 knockdown [5]. Previous studies have also shown that MDM2 can ubiquitinate and degrade p73, albeit to a less extend compared to MDM2-mediated p53 ubiquitination and degradation. Interestingly, the level of p73 is still reduced by RPL26 knockdown in MDM2 knockout cell lines, although this effect is less robust compared to that in MDM2-proficient cells. Polysome profiling and translation analyses clearly showed that knockdown of RPL26 reduced p73 translation independently of MDM2 and p53 [5]. An elegant set of *in vitro* and in cell analyses showed that unlike its regulation of p53 translation, RPL26 specifically binds to the 3’-UTR, but not 5’-UTR, of p73 mRNA and robustly promote p73 translation [5]. Further, RPL26 directly binds to eIF4E, a cap-binding protein in the translation initiation factor eIF4F complex that also includes eIF4A and eIF4G. Ablation of eIF4E abolished the regulation of p73 by RPL26 [5], suggesting that RPL26 promotes p73 translation initiation. Of note, RPL26 regulation of p73 may still involve the 5’-UTR and 3’-UTR looping in the absence of dsRNA formation, as it interacts with the eIF4E initiation complex, in which eIF4G binds to the Poly(A)-binding protein (PABP) to promote cap-dependent translation (Figure 1). Functionally, knockdown of RPL26 promotes whereas overexpression of RPL26 suppresses cell proliferation in a p73-dependent and p53-independent manners. Together, this study provides two distinct mechanisms by which RPL26 regulates the expression of p73: stabilizing p73 by inhibiting MDM2 and promoting p73 translation by binding to p73 mRNA 3’-UTR and eIF4E (Figure 1). The dual mechanisms ensure that p73 is induced in cells in response to ribosomal stress to tightly coordinate ribosome biogenesis with cell cycle progression in the absence of p53.

Further supporting this RP regulation of p73 is that RPL5 and RPL11 have recently been shown to bind to MDM2 to regulate p73 stability [8]. Hence, it is likely that a subset of RPs regulate the levels and activity of both p53 and p73 in response to ribosomal stress. It is interesting to examine how the RP-p53 and the RP-p73 axes tightly co-regulate cell growth and proliferation in normal cells and how the RP-p73 axis functions in cancer cells with p53 deletion or mutations. It is also interesting to test how RPL26 interacts with the eIF4F complex and PABP in translation initiation and whether and how RPL26 association with these proteins and p73 mRNA is regulated (e.g. posttranslational modifications) following ribosomal stress. Further defining mRNA sequence and structure at the p73 mRNA 3’-UTR mediating RPL26 binding could elicit important mechanistic information about p73 translation regulation in normal and cancer cells. Finally, future studies are warranted to examine whether RPL26 regulates other tumor suppressor mRNAs and whether other RPs can regulate the translation of p73 and p53 as well.
